# Supplementary material for: Oral Immunization with Yeast-Surface Display of SARS-CoV-2 Antigens in Pichia pastoris Induces Humoral Responses in BALB/C Mice
Source: Infect Dis Rep. 2025 Aug 27;17(5):104. doi: 10.3390/idr17050104 (PMC12452405; doi:10.3390/idr17050104)
Supplement: Supplementary file 1 [file idr-17-00104-s001.zip › idr-3676279-supplementary.pdf]

Supplementary Materials

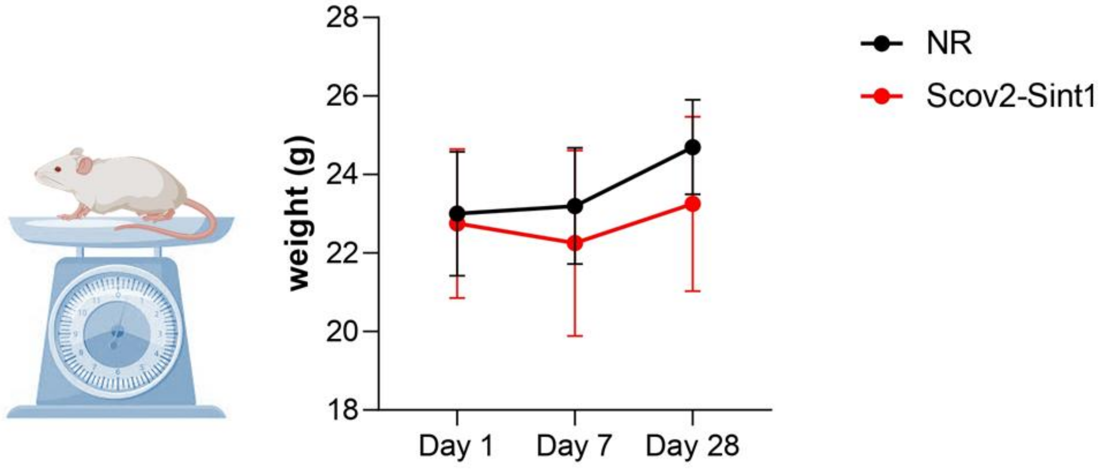

Figure S1. Average weight of groups of mice during the immunization schedule. Values correspond to mean ± standard deviation. NR: Non-recombinant.

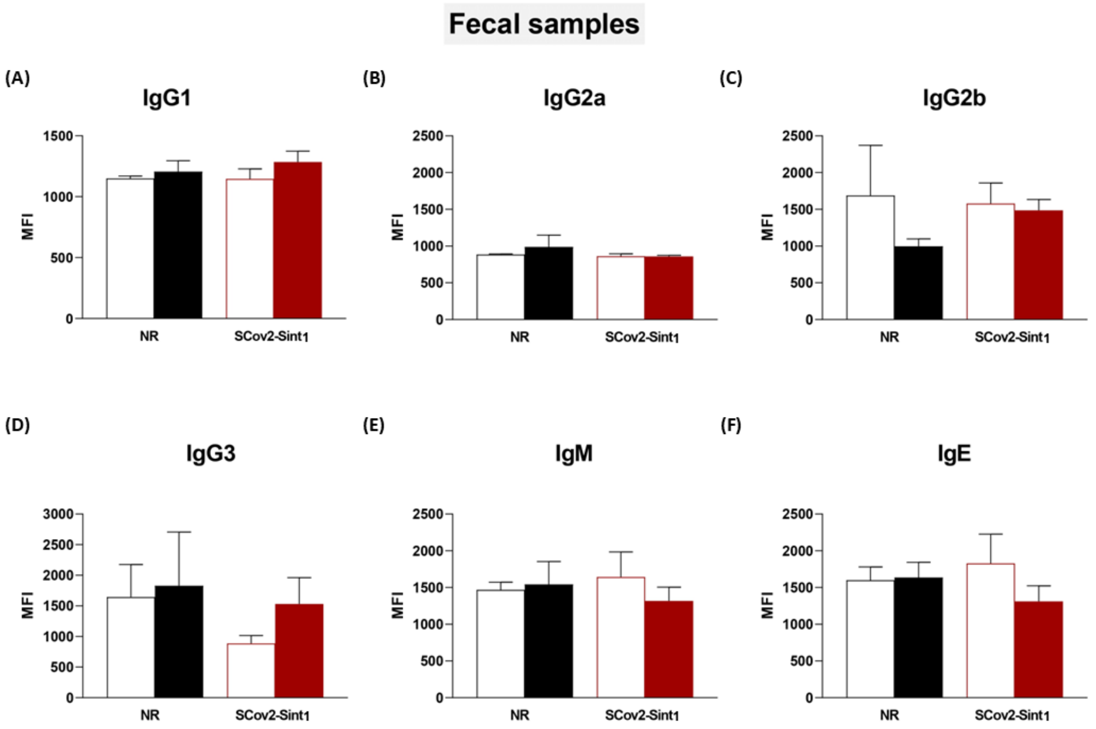

Figure S2. Evaluation of immunoglobulin isotypes detected in the feces of animals after the immunizations. The values correspond to the samples collected 7 days after the first dose (white bars) and 21 days after the second dose (colorful bars). (A) IgG1, (B) IgG2a, (C) IgG2b, (D) IgG3, (E) IgM, and (F) IgE. MFI corresponds to the mean fluorescence intensity. Bars indicate the mean value ± standard deviation.

Table S1. Differential leukocyte counts between vaccine groups: G1) *P. pastoris* GS115 (non-recombinant); and G2) *P. pastoris* Scov-Sint1. RV: Reference value. Values correspond to the mean ± standard deviation.

	**G1**	**G2**	**RV** [35,36]
**Lymphocytes**	80,20 ± 4,28	83,63 ± 4,73	71.76 ± 5.9
**Neutrophils**	14,80 ± 4,07	14,00 ± 4,92	22.96 ± 5.54
**Monocytes**	3,50 ± 1,41	2,13 ± 0,95	2.68 ± 1
**Eosinophils**	2 ± 2	1 ± 1,15	2.16 ± 1.71
**Basophils**	0 ± 0	0 ± 0	0 ± 0

References

35. Barbosa, B.D.S.; Praxedes, É.A.; Lima, M.A.; Pimentel, M.M.L.; Santos, F.A.; Brito, P.D.; Lelis, I.C.N.G.; Macedo, M.F.D.; Bezerra, M.B. Haematological and Biochemical Profile of Balb-c Mice. *Acta Scientiae. Vet.* **2017**, *45*, 5. https://doi.org/10.22456/1679-9216.80473.
36. Silva-Santana, G.; Bax, J.C.; Fernandes, D.C.S.; Bacellar, D.T.L.; Hooper, C.; Dias, A.A.S.O.; Silva, C.B.; De Souza, A.M.; Ramos, S.; Santos, R.A.; et al. Clinical Hematological and Biochemical Parameters in Swiss, BALB/c, C57BL/6 and B6D2F1 *Mus. Musculus*. *Anim. Models Exp. Med.* **2020**, *3*, 304–315. https://doi.org/10.1002/ame2.12139.
